# Natural Ingredients from Medicine Food Homology as Chemopreventive Reagents against Type 2 Diabetes Mellitus by Modulating Gut Microbiota Homoeostasis

**DOI:** 10.3390/molecules26226934

**Published:** 2021-11-17

**Authors:** Xiaoyan Xia, Jiao Xiao

**Affiliations:** 1 School of Traditional Chinese Medicine, Shanxi Datong University, Datong 037009, China; xiaoyanxia1202@163.com; 2 Wuya College of Innovation, Shenyang Pharmaceutical University, Shenyang 110016, China

**Keywords:** medicine food homology, functional food herbs, type 2 diabetes mellitus, gut microbiota

## Abstract

Type 2 diabetes mellitus (T2DM) is a noteworthy worldwide public health problem. It represents a complex metabolic disorder, mainly characterized as hyperglycemia and lipid dysfunction. The gut microbiota dysbiosis has been proposed to play a role in the development of diabetes. Recently, there has been considerable interest in the use of medicine food homology (MFH) and functional food herbs (FF) to ameliorate diabetes and lead to a natural and healthy life. Hence, this review compiles some reports and findings to demonstrate that the practical use of the MFH/FF can modulate the homoeostasis of gut microbiota, thereby ameliorating the development of T2DM. The results provided useful data to support further investigation of the functional basis and application of MFH/FF to treat T2DM through maintaining intestinal homeostasis.

## 1. Introduction

Diabetes mellitus is one of the major public health problems and has become a health global burden. Based on the data of IDF, there were approximately 451 million diabetic patients aged 18 to 99 in the world in 2017. By 2045, this figure is expected to increase to 693 million [1]. Diabetes mainly includes Type-1 (T1DM) and Type-2 Diabetes Mellitus (T2DM), of which T2DM accounts for roughly 90–95% [2]. T2DM is a complex metabolic disorder, which is chiefly characterized by hyperglycemia, with glycolipid dysfunction, progressive loss and dysfunction of islet β-cell, and insulin resistance. T2DM is usually accompanied by oxidative stress and inflammation, and long-term hyperglycemia may lead to diverse diabetic complications [3].

One of the main reasons for the sharp increase in the incidence of T2DM is the significant changes in human behavior and lifestyle. Through diet modification, regular exercise, weight control, and patient education, T2DM can be managed and medications can be avoided. Diabetes is characterized by “leaky gut” syndrome, where bacterial cell wall components enter the blood circulation of the animal host in a large amount, which may cause metabolic endotoxemia and systemic low-grade inflammation [4]. Gut microbiota acts an important role in modulating the systemic and intestinal immunity and metabolic homeostasis [5]. Studies have shown that the consortium of gut microbiota is closely related to host genetics and other diverse conditions, such as food habits, stresses, exposure to drugs or toxins [5]. It was reported that the gut microbiota in healthy people is diversified, achieving more short chain fatty acids (SCFA) and producing more branched amino acids, while the intestinal flora of diabetes is more likely to produce compounds that affects glucose metabolism. The intestinal microbiota can digest diverse dietary fibers that cannot be digested by the host, and produce SCFAs as its metabolites, such as acetate, butyrate and propionate [6]. Propionate can maintain gluconeogenesis in the intestinal tract, thereby making better use of energy, while butyrate, with anti-inflammatory activity, can reduce the permeability of the intestine [6]. In T2DM patients, butyrate producing microbiota are significantly reduced, specifically the Clostridiales order, including the genera *Ruminococcus* and *Subdoligranulum*, and species such as *Roseburia intestinalis* and *Roseburia inulinivorans* [7,8].

In ancient China, diabetes was called ‘Xiao Ke’, manifested as persistent thirst and hunger, excessive urination, and weight loss. For thousands of years, Chinese herbal prescriptions and traditional Chinese medicine (TCM) medicinal materials have been commonly used to intervene in ‘Xiaoke’ disease. Historically, many of the formulations and medicinal herbs have been used as food for safe and effective long-term consumption [9]. Natural plants are essential for the management of many human diseases, such as diabetes [10]. Numerous herbal medicinal plants are natural sources of antioxidants, which can reduce the oxidative stress generated by STZ in β-cells. World Health Organization (WHO) has recommended the evaluation and application of traditional botanical treatments for diabetes because they are effective and non-toxic, have fewer side effects or have no side effects, and are considered excellent candidates for oral therapy [11]. In recent years, more and more researchers have been paying attention to natural products from traditional herbs and foods for their safety, efficacy, and potency in treating diabetes [12].

The concept of ‘medicine and food homology’ was proposed in the *Huang Di Nei Jing Su Wen*: ‘Eating on an empty stomach as food, and administering to the patient as medication’ embodies the theory of medicine food homology (MFH); that is, some food classes can also be used as drugs. Functional food (FF), also known as health food, refers to a specific type of food that is not aimed at curing diseases but can modulate human body functions. “Notice on Further Regulating the Management of Raw Materials for Health Foods” was issued in 2012 by the Ministry of Health, which covers both foods and medicines [9]. In addition, 110 MHF and 114 FF are currently included in this promulgated management method. More and more clinical evidence clarifies that the occurrence and development of T2DM can be prevented or delayed by regular intake of foods that are believed to be functional and affect glycemic control, antioxidant enzymes activity and intestinal flora, while also inhibiting the excessive production of pro-inflammatory cytokines during diabetes [13].

**Figure 1:**
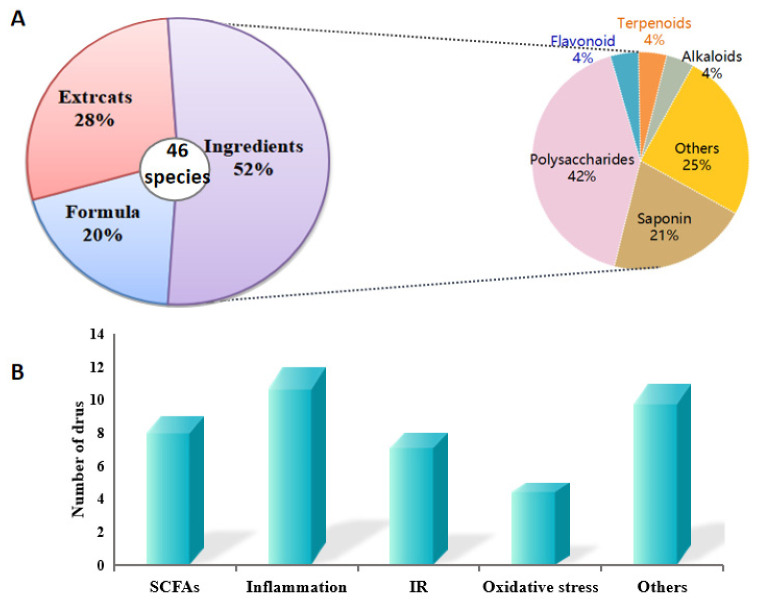
Analysis of MFH and FF showing anti-diabetic effects by modulating gut microbiota in this review. (**A**) Classification about the active MFH and FF species. (**B**) the relational mechanism of MFH and FF on T2DM associated with gut micorobiota.

Therefore, in this review, we searched various online databases (PubMed, ScienceDirect, CNKI) and scientific publications from the library using qualitative systematic reviews. The review was based on MHF and FF application for possessing medicinal value against T2DM by modulating the gut microbiota (Figure 1A).

## 2. Association between Gut Microbiota and T2DM

### 2.1. Alteration of Gut Microbiota Composition with T2DM

Although T2DM is caused due to various factors, the human gut microbiota plays a vital role in the progression of T2DM [4]. The impact of gut microbiota on T2DM has attracted widespread attention; studies have been done over the past few years to research the relationship between the two [12]. ‘Human microbiome’ was firstly defined by Joshua Lederberg in 2001 as an ecological community of symbiotic and pathogenic microorganisms that share our body space. An adult is colonized by almost 100 trillion microbes, which mainly exist in the gastrointestinal tract, with the largest group living in the colon. Human health is strongly affected by the microbiota that coexist with our body [14]. The diversity of the intestinal flora of T2DM patients is significantly decreased compared to that of healthy controls [15,16]. As Larson et al. reported, the abundance of Firmicutes phylum in diabetic patients was reduced when compared to non-diabetic patients, and the ratio of Bacteroides to Firmicutes was positively correlated with blood glucose levels [17].

### 2.2. Mechanism of Gut Microbiota Alteration Causing T2DM

T2DM, characterized by “leaky gut” syndrome, is known to have markedly enhanced intestinal permeability, allowing bacteria to translocate across the intestinal epithelium, resulting in host metabolic endotoxemia and triggering low-grade inflammation. In T2DM, the abundance and diversity of the gut microbiota both decreased, accompanied by an increase in the abundance of pathogenic microorganisms and a decrease in the abundance of symbiotic microorganisms [18]. For example, the generas *Faecalibacterium*, *Roseburia* and *Bifdobacterium*, with noticeable abilities to reduce intestinal permeability, have been shown to be exhausted in T2DM [19]. The changes in the above-mentioned microbiota would lead to low-grade inflammation, resulting in a decrease in mucus layer and disintegration of the epithelial membrane. This is followed by an increase in intestinal permeability, allowing lipopolysaccharides (LPS) to enter the blood circulation. Bacterial fragments and LPS can be recognized by innate toll-like receptors (TLRs), particularly TLR-4, which subsequently stimulates the activation of transcription factor κB (NF-κB) and the release of pro-inflammatory mediators in intracellular signaling pathways [20]. The release of pro-inflammatory cytokines would further result in the destruction of glucose metabolism and insulin signaling pathways [21]. Metabolic endotoxemia and low-grade inflammation occurs, subsequently. The systemic low-grade inflammation affects all vital organs or tissues, such as the pancreas, liver, and kidney [22]. For example, the level of TNF-α in T2DM is significantly increased, which is closely related to islet dysfunction [16,23]. Under this circumstance, the homeostasis of glucose metabolism no longer exists and thus results in type 2 diabetes. In this condition, the steady state of glucose metabolism collapses and T2DM is developed [24].

## 3. Bioactive Ingredients of MFH and FF Target for Microbiota in T2DM

**Figure 2:**
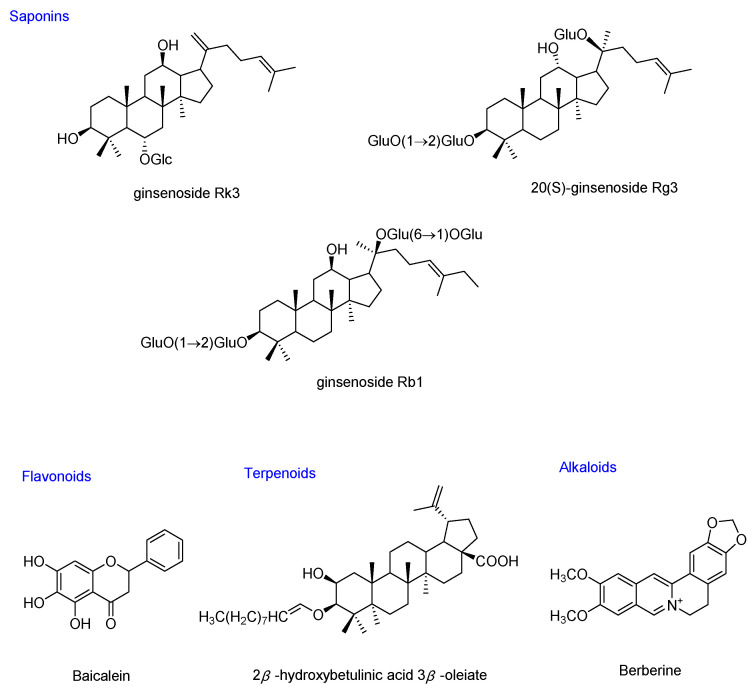
Chemical structures of the representative hypoglycemic compounds from MFH and FF that can modulate gut microbiota in T2DM.

The diet and its metabolites have a major physiological impact on the composition of gut microbiota and the health of the host [22]. In China, MFH and FF refers to a group of foods that can also be used as medicines, many of which possessed anti-hyperglycemic activities. Regular consumption of MFH, which is considered to affect glycemic control, activation of antioxidant enzymes and gut microbiota, and to inhibit the excessive production of pro-inflammatory cytokines, to prevent or treat T2DM [25]. MFH and FF have been widely sought recently, and research into their use for T2DM has evoked considerable interest. Herein, the bioactive ingredients of MFH and MHF were divided into: saponins, polysaccharides, flavonoids, terpenoids, alkaloids, and others, and their anti-diabetic effects via gut miocrobiota regulation were listed in Table 1, and the chemical structures of the representative compounds are shown in Figure 2.

### 3.1. Saponins

Saponins are a class of glycosides composed of triterpenes or spirostanes, which are widely present in nature [52]. Saponins have been reported to have a wide range of hypoglycemic targets and pathways, which can directly repair damaged islet cells and increase insulin levels to maintain normal blood sugar. Saponins can also regulate blood lipids and improve glucose tolerance. This suggests that they have broad research and development prospects as anti-diabetic drugs [53]. In this review, saponins from *Panax ginseng*, Panax notoginseng, Korean red ginseng and Polygonatum sibiricum were researched. It was observed that these saponins can intervene on T2DM and are associated with their modulating the imbalance of gut microbiota and inhibiting the low-grade inflammation and insulin resistance.

Ginseng, a perennial herb of the genus Panax, has been used widely as a TCM herbs in China and Asia for thousands of years. About 4000 years ago, “Shen Nong Ben Cao Jing” is the earliest surviving TCM monograph in China. It records the use of ginseng as a health medicine to delay aging and nourish the body without side effects [54]. According to Zhang Zhongjing’s Shang Han Za Bing Lun in the Han Dynasty, ginseng was used to cure thirst, which is the main symptom of “Xiaoke” (diabetes). Additionally, the “Tai Ping Hui Min He Ji Ju Fang”, an official traditional Chinese medicine book in Song Dynasty, recorded the use of ginseng to treat “Xiaoke disease”. Many of the Chinese patent medicines approved by the government for the treatment of diabetes contain ginsenosides, such as Tianqi capsules [55], Jinlida Granule [56], and ShenMai Injection [28,57]. Ginseng has several therapeutic functions, such as anti-stress, maintaining and strengthening the central and immune system, preventing certain chronic diseases, and delaying aging. While American ginseng is more effective in treating cardiovascular disease [58]. Ginsenosides are extracted from the roots and rhizomes of *Panax ginseng* C. A. Meyer. Research has shown that ginsenosides show noticeable anti-diabetic activities and have been used as adjuvants for diabetes treatment in China. It was reported that saponins isolated from ginseng, such as Ginsenoside Rk3, and 20(S)-ginsenoside Rg3 showed potential anti-diabetic activities by regulation of gut microbiota.

It was observed that Ginsenoside Rk3 at the dose of 30 and 60 mg/kg/day could alleviate the abundance imbalance of gut microbiota and inhibit the expression of pro-inflammatory cytokines by reducing intestinal permeability and LPS levels, thereby preventing low-grade colon inflammation caused by a high-fat diet in mice. An 8-week intervention of Rk3 could significantly decrease the ratio of Firmicute to Bacteroidete, and restore the abundance of Lactobacillaceae, Helicobacteraceae, Neococcaceae, and Bifidobacteriaceae at the dose of 60 mg/kg/day. Ginsenoside Rk3 can effectively improve the C57BL/6 Mice metabolic disorder of gut microbiota by decreasing the ratio of Firmicute/Bacteroidete, and inhibit the inflammatory cascade by suppressing the TLR4/NF-κB pathway [26]. It was found that 20(S)-ginsenoside Rg3 at a dose of 20 mg/kg can reduce the blood glucose by regulating the metabolism of gut flora in T2DM rats [27].

Polygonatum sibiricum, a perennial herb of Liliaceae family, has diverse activities, such as hypoglycemic effect, regulating blood lipids, delaying aging, and strengthening immunity. A saponin was isolated from P. sibiricum (PSS) and administered to diabetic mice at the dose of 1.0, 1.5, or 2.0 g/kg/day. It was found that PSS could alleviate the symptoms of polyphagia and polydipsia and regulate the gut microbiota in the diabetic mice. PPS increased the abundance of probiotics (including Bifidobacteria and Lactobacillus), and down-regulated the harmful bacteria (such as Enterobacteriaceae, Enterococcus, and C. perfringens) [30].

### 3.2. Polysaccharides

Polysaccharides are formed by the polymerization of monosaccharide molecules through glycosidic bonds, which are generally composed of hundreds or even thousands of monosaccharides molecules with a relatively high molecular weight. Polysaccharides, as a kind of abundant natural product, are found in organisms such as fungi and plant roots [59]. As prebiotics, polysaccharides have been found to affect the populations and metabolism of the gut microbiota, and attracted widespread attention in biochemical and medical research [60]. The polysaccharides from MFH have been studied to show potential impact on T2DM, which is associated with the regulation of gut microbiota.

Ophiopogonis Radixa, the Chinese name Maidong, is the tuberous roots of *Ophiopogon japonicus* (Thunb.) Ker-Gawl (Liliaceae), which is a popular TCM. Maidong is widely used as a functional food in China. Maidong has been used to relieve diabetes and cardiovascular diseases for years [31]. The polysaccharide is one of the main active ingredients of Maidong. A homogeneous polysaccharide fraction was isolated and characterized from Ophiopogonis Radix collected from Sichuan. Ophiopogonis Radix and was analyzed for anti-diabetic effects in targeting β-cell dysfunction, insulin enhancement and inhibiting α-amylase and α-glucosidase [61]. The anti-diabetic effects of polysaccharides, isolated from Maidong, have been reported [62,63]. More than 15 kinds of polysaccharides have been isolated from Maidong, which show a good anti-diabetic effect, and the main mechanism is associated with improving β-cell dysfunction, enhancing insulin and inhibiting α-glucosidase and α-amylase [64]. For example, MDG-1, a kind of polysaccharide isolated from Maidong, possessed anti-diabetic effects in diabetic mice and regulated intestinal flora in obese mice [31,64]. In KKay mice, the abundance of Escherichia coli and Streptococcus increased, while the abundance of Lactobacillus and Bifidobacterium decreased. However, oral administration of 300 mg/kg MDG-1 can reduce the number of pathogenic *E. coli* and Streptococcus, and increase the number of Lactobacillus (*p* < 0.05). It has been proven that oral MDG-1 can improve the glucose tolerance of diabetic mice and is related to its regulating effect on the intestinal microecological balance [32].

*Lycium barbarum* L. and its mature fruits have been used as a TCM and functional food in China for about 2000 years. The leaves of *L. barbarum*, also named Tianjing grass, are widely used as tea, food and medicine in China due to its activities of reinforcing deficiency and benefiting essence, as well as anti-thermic and eye-clearing effects [65,66]. An HFD/STZ-induced T2DM rat model was established to study the anti-diabetic effects of the water extract of *L. barbarum* leaf (LLB). It was found that LLB can improve T2DM, which is mainly associated with the reversal of gut microbiota imbalance, and regulation of nicotinate/nicotinamide, arachidonic acid/purine metabolism. Administration of LLB at 2.08 g/kg T2DM rats significantly reduced excessive abundance of Parasutterella, Marvinbryantia, Blautia, Ruminococcus_1, and Prevotellaceae_NK3B31_group, and reversed the ratio of Firmicutes to Bacteroidetes in the gut microbiota of diabetic rats [66]. The homogeneous polysaccharide (LBP-W) was purified from crude *Lycium barbarum* polysaccharides (LBPs), and administration of 50 mg/kg LBP-W could improve obesity by modulating the composition of gut microbiota and the metabolism of SCFAs in C57BL/6 mice on a high fat diet. LBP-W intervention reversed the HFD-induced changes in Firmicutes and Bacteroides, and the ratio of Firmicutes/Bacteroides was noticeably reduced (*p* < 0.01) [33].

*Plantago asiatica* L. is a kind of TCM and has been used as a folk medicine worldwide [67,68]. A high-fat diet and STZ induced T2DM rat model has been established, and the anti-diabetic effect of *Plantago asiatica* L. polysaccharide (PLP) was studied. It was observed that administration of PLP (at dose of 100, 200 or 400 mg/kg) significantly decreased the level of blood glucose, insulin, serum lipids, non-esterifified fatty acid and maleic dialdehyde, and noticeably increased the activities of antioxidant enzymes in T2DM rats after 4 weeks of PLP intervention. The concentrations of SCFA were noticeably higher in the feces of diabetic rats after treating with PLP. Moreover, colon bacterial diversity and abundance of bacteria, including Bacteroides vulgatus, Lactobacillus fermentum, Prevotella loescheii and Bacteroides vulgates were markedly increased by PLP intervention. It indicated that the anti-diabetic effect of PLP inT2DM rats was related to the regulation of gut microbiota and increased levels of SCFAs production [37].

*Apocynum venetum* is a perennial herbaceous or half-shrub plant, and its leaves have been traditionally consumed as a tea beverage in China. *A. venetum* is widely distributed in saline-alkali land, riverbanks, fluvial plains and sandy soils of Asia and North America [69]. Hypoglycemic and hypolipidemic effects of polysaccharide-rich extracts from *A. venetum* leaves on T2DM mice has been studied. Treatment of alkaline extracted polysaccharide-rich products markedly decreased the levels of fasting blood glucose, serum insulin, and serum lipids. Meanwhile, the reduced glycogen contents in liver were prominently improved, and the oxidative damage was markedly ameliorated by alkaline extracted polysaccharide products in diabetic mice. Furthermore, the polysaccharide-rich extracts could reverse the gut microbiota dysbiosis in T2DM mice by increasing the abundance of genera Odoribacter, Anaeroplasma, Parasutterella, and Muribaculum, while decreasing the abundance of genera Enterococcus, Klebsiella, and Aerococcus. Thus, polysaccharide-rich extracts of *A. venetum* showed good anti-diabetic effects for treating T2DM, which was associated with the intervention of gut microbiota [35].

In this review, polysaccharides from MFH and FF were summarized and their impacts on T2DM by regulating gut microbiota were listed in Table 1. It was found that the polysaccharides play an important role in maintaining intestinal flora steady state, which was associated with the promotion of short-chain fatty acids (SCFAs). SCFA mainly include acetate, butyrate and propionate at the ratio of 3:1:1 in human gut microbiota, which are usually present in the human intestine at a ratio of 3:1:1 and are in a steady state [70]. Butyrate possesses anti-inflammatory effects and can reduce intestinal permeability, and propionate also maintains gluconeogenesis in the intestines, thereby making better use of energy [24]. Individuals with T2DM have reduced butyrate-producing gut microbiota, which promotes low-grade inflammation [70].

### 3.3. Flavonoids

Flavonoids are meaningful natural compounds that exist widely in the plant kingdom and have a basic 2-phenyl-chromone structure. They are a class of secondary plant compound with noticeable physiological effects and various health benefits [9]. Flavonoids possess extensive pharmacological effects, among which are antioxidant and free radical scavenging activities, which are of particular interest to the pharmaceutical industry [71]. Flavonoids are widely reported to prevent and treat T2DM by affecting the function of islet β-cells and anti-lipid peroxidation [72]. However, not so many flavonoids from natural herbs were found to intervene T2DM by the regulation of gut microbiota.

Baicalein is a dietary flavonoid and is a main component of Oroxylum indicum and Scutellaria baicalensis. It is used as a dietary supplement or as tea in Asia, Europe and the Americas. Based on Zhang’s study in 2018, four weeks intervention of baicalein (50, 150 mg/kg·d) significantly decreased the blood glucose and LPS and improved insulin resistance, inflammation, and lipid profile in T2DM rat dose-developmentally. These anti-diabetic effects are owing to the increase in SCFAs content and the thickness of the intestinal mucus layer, which is closely related to the regulation of the intestinal microbiota, especially the abundance of Bacteroides and Bacteroides S24-7. They had the highest relative abundance in rats receiving 150 mg/kg baicalein, and they were positively correlated with improving T2DM-related phenotypes [42]. As reported, Bacteroidales S24-7, Prevotella, Blautia, and Butyricoccus are the key SCFA-producing bacteria, which may relieve inflammation and insulin resistance, by reducing the intestinal endotoxins entering the circulation, thereby alleviating T2DM [73,74].

Plumula nelumbinis, also named “Lian-Zi-Xin” in Chinese, is the dried embryo of the ripe seeds of Nelumbo nucifera Gaertn (Nelumbonaceae). It is a traditional Chinese medicine (TCM), and also an ordinary health food. It is commonly used in several counties around the world. In TCM, Lian-Zi-Xin has been used to clear heart heat, calm the mind, and treat high fever, promote astringent essence and hemostasis [75,76]. As Qiuzhe Li reported in 2015, the total flavonoids from Lotus plumule showed noticeable anti-diabetic effects by reducing the blood glucose level, regulating blood lipid levels and improving the glucose tolerance in the T2DM mice.

### 3.4. Terpenoids

Terpenes are natural hydrocarbons and can be linked in diverse ways through isoprene or isopentane. It mainly includes monoterpenes, sesquiterpenes, diterpenes and triterpenes, which play a vital role in organisms. Studies have shown that some terpenoids possess a preventive effect on T2DM; the mechanism may be mediated by protecting islet β-cells and increasing glucose tolerance and hepatic glycogen synthesis [71]. However, there are few studies on the effects of terpenoids treating T2DM by regulating gut microbiota.

A pentacyclic triterpene, 2β-hydroxybetulinic acid 3β-oleiate (HBAO), was isolated from the seeds of Euryale ferox salisb. Oral administration of 60 mg/kg/d HBAO could ameliorate glycemic homeostasis and alleviate oxidative stress in the streptozocin (STZ)-induced diabetic rats. It was observed that HBAO normalized the blood glucose, glycosylated hemoglobin (HbA1c), hepatic hexokinase and plasma insulin, improved damaged pancreatic β-cell, regulated dyslipidemia and antioxidant enzymes (such as superoxide dismutase, catalase and glutathione peroxidase) in the diabetic rats (*p* < 0.05) [43]. STZ-induced diabetic mice were administrated by the triterpenoid-rich extracts of Euryale ferox shell (ES) orally at doses of 200, 300, 400, 500 ± 2 mg/L for 4 weeks. It was found that the triterpenoid-rich extracts of ES could regulate glucose metabolism (*p* < 0.01), normalize the body weight of the diabetic mice (*p* < 0.01), reduce the expression of the negative regulation protein PTP1B gene and increase insulin receptor IRS-1 protein expression (*p* < 0.05) [77].

### 3.5. Alkaloids

Alkaloids are a class of nitrogen-containing organic compounds derived in nature, mainly in the plant kingdom. Most alkaloids are alkaline and have significant biological activity and are a kind of important bioactive ingredient in MFH and FF [71]. It has been found that the hypoglycemic activities of alkaloids are mainly mediated by inhibition of gluconeogenesis, regulation of gut microbiota structure, promotion of glycolysis and anti-glucagon activities, promotion of the secretion of pancreatic β-cells, and scavenging of oxygen free radicals [78]. For example, neferine could reduce the levels of blood glucose, improve insulin resistance and regulate the disorder of lipid metabolism in T2DM rats [75]. Isoliensinine was found to attenuate T2DM with hyperlipidemia in a KK-Ay mouse model by regulating GLUT4, SREBP-1c, PPARγ, AMPK and ACC phosphorylation [76]. However, there are few studies on the effects of alkaloids treating T2DM through gut microbiota regulation.

Berberine is an isoquinoline quaternary alkaloid that is widely found in Coptidis rhizoma and Berberis vulgaris [79]. Berberine has a long history in Chinese and Western medicine treatment [80]. In China, Berberine has been used to treat diarrhea caused by bacteria as an over-the-counter drug for years [45,81]. Berberine was administrated to T2DM rats, and it was found that the anti-diabetic effects of Berberine is related to its regulation of gut microbiota. The community richness and diversity of the gut microbiota were noticeably increased by Berberine, and the abundance of Bacteroidetes was increased, while the number of Proteobacteria and Verrucomicrobia were decreased. At the family level, a probiotic Lactobacillaceae was markedly increased after Berberine intervention, which was negatively related to the risk of T2DM [44]. It suggested that Berberine can alleviate T2DM in rats by modulating gut microbiota composition.

### 3.6. Others

Some other kinds of compounds in medical herbs, such as proteins, fibers, essential oil and glycosides, also show significant hypoglycemic activities. We also researched and summarized those kinds of ingredients from MFH and FF to find the potential anti-diabetic compounds.

*Rehmannia glutinosa* is a kind of perennial herbaceous plant of the *Scrophulariaceae* family. The *R. glutinosa* leaves’ total glycoside (DHY) is mainly composed of iridoid glycosides and phenylethanoid glycosides extracted from *R. glutinosa* leaves. Studies have shown that DHY has been used in the clinical treatment of various kidney diseases, due to its protection on kidneys by improving glomerular permeability and reducing proteinuria. DHY was also found to improve STZ-induced gut microbiota imbalance in diabetic nephropathy rats [46]. DHY was observed to significantly decrease the levels of blood glucose, serum lipid (such as total cholesterol and triglyceride) and improve kidney damage, and inhibit the expression of α-SMA, TGF- β1, Smad3 and Smad4 in the kidney tissues of *db/db* mice. DHY had noticeable up-regulation effect on *Firmicutes* in *db/db* mice. At the genus level, DHY were dominant for the recovery of *norank_f_Bacteroidales_S24_7_group* in *db/db* mice. Therefore, DHY may restore the dysfunctional intestinal flora to normal and regulate glycolipid level of *db*/*db* mice [46].

*Salvia miltiorrhiza* Bge., a TCM for promoting blood circulation and removing blood stasis, has been used as a health-care food recently. The aerial parts of *S. miltiorrhiza* Bge. (DJ) are rich in phenolic acids similar to the rhizome [82]. The 60% ethanol extracts of DJ were found to strengthen the intestinal barrier of diabetic mice by up-regulating the tight junction proteins expressions in ileum and colon, but not in duodenum. DJ could modulate the diabetes-induced gut microbiota imbalance. At phylum level, that the number of *Proteobacteria* was significantly increased while *Tenericutes* was significantly decreased in DJ group compared to the control group [82].

Dietary fibers can modify the gut barrier and microbiota homeostasis, thereby impacting the progression of diabetes. Inulin-type fructans (ITFs) are natural soluble dietary fibers with different fermentation degrees in chicory root, which can regulate the occurrence and development of diabetes. Female nonobese diabetic mice were weaned to long-and short-chain ITFs, ITF(l) and ITF(s) supplemented diet up to 24 weeks. Expression of barrier reinforcing tight junction proteins occludin and claudin-2, antimicrobial peptides-defensin-1, and cathelicidin-related antimicrobial peptide as well as short-chain fatty acid production were enhanced by ITF(l). It was found that ITF(l) enhanced *Firmicutes*/*Bacteroidetes* ratio to an antidiabetogenic balance and enriched modulatory *Ruminococcaceae* and *Lactobacilli* [49]. The inulin was found to alleviate different stages of T2DM in diabetic mice by modulating gut microbiota. It increased the relative abundance of *Cyanobacteria* and *Bacteroides*, and reduced the relative abundance of *Deferribacteres* and *Tenericutes*. Dietary inulin can ameliorate diverse stages of T2DM by suppressing inflammation and modulating gut microbiota, especially in pre-diabetic and early diabetic stages, thus it potentially serves as an inexpensive intervention for the prevention and treatment of T2DM patients [15].

## 4. Herb Extracts of MFH and FF Target for Microbiota in T2DM

### 4.1. Single Herb Extracts of MFH and FF Target for Microbiota in T2DM

Generally, MFH and FF are usually taken in the form of decoction or direct consumption. Therefore, the anti-diabetic effect of the water extracts or total extracts is worthy of attention. We investigated the anti-diabetic effects of the extracts of MFH and FF by regulating the imbalance of the intestinal microbiota, such as *Fructus Aurantii Immaturus*, *Atractylodis macrocephalae Rhizoma*, Radix *Puerariae*, *sea buckthorn*, *Anemarrhena asphodeloides*, *Dendrobium officinale*, listed in Table 2.

*Atractylodis macrocephalae* Rhizoma is widely used as a functional food in Asia. The water extracts of *A. macrocephalae* (AMK) at a dose of 100 mg/kg noticeably increased the relative abundance of *Bacteroides thetaiotaomicron* and *Methanobrevibacter smithii* in gut microbiota of the diabetic mice. It was found that AMK could significantly decrease the blood glucose and serum lipids, and improve the insulin resistance, which was associated with its inhibitory effects on inflammation and its regulation of gut miocrobiota imbalance.

Chinese propolis, is a resinous substance collected by bees from plants exudates that is mixed with wax and mandibular gland secretions [96]. Propolis has long been recognized as a natural nutraceutical has shown a beneficial effect on alleviating by exerting good anti-inflammatory, anti-oxidant effects [96]. Studies have reported that propolis extract could boost lipid metabolism, alleviate insulin resistance, and delay obesity in high-fat diet-fed mice and rats with T2DM [87]. Propolis were abserved to reverse the elevation of *Firmicutes* and inflammatory biomarkers expression induced by HFD in the obese mice [88]. Propolis intervention can regulate gut microbiota by decreasing *Alistipes*, and increasing *Lactobacillus* in male mice, which are playing an important role in the preventive effect on obesity and T2DM.

### 4.2. Herb Formula Consisted of MFH for T2DM by Regulating Microbiota

Chinese herbal formulas with anti-diabetic effects have been well studied, and many of them have commonly been used in “Xiaoke” patients since ancient times. In the traditional Chinese medicine system, the relationship between the gut microbiota and disease is actually the relationship between the intestine and disease, which was early mentioned in the “*Huang Di Nei Jing*”. Therefore, we summarized the herb formulas consisted of MFH, which act anti-diabetic effects by regulating the imbalance of gut microbiota, and listed in Table 3.

Wumei Wan was first recorded in Zhang Zhongjing’s “*Shanghan lun*”, and is the main prescription for the treatment of Jueyin disease. “Xiaoke” disease was considered to be one of Jueyin diseases as recorded in ancient China. It was found that Wumei Wan (at the dose of 20, 10, 5 g/kg/d) could significantly enrich the functional bacteria, such as *Firmicutes*, *DeltaProteobacteria*, and *Lactobacillus*, and decrease the abundance of *Bacteroidetes*, *Actinobacteria*, *Bacteroides*, *Clostridium* in the T2DM rats [97]. It has been proven that Wumei Pill can regulate the balance of intestinal flora in T2DM model rats, increase the content of short-chain fatty acids (including acetic acid, propionic acid, butyric acid), thereby lowering blood glucose and ameliorating T2DM. Daesiho-Tang is another important formulation in TCM, known for its anti-diabetic and anti-hepatotoxic effects. It has been found that Daesiho-Tang treatment noticeably increased the relative abundance of *Bacteroidetes*, *Bacteroidetes*/*Firmicutes* ratio, *Akkermansia Bifidobacterium*, *Lactobacillus*, and decreased the level of *Firmicutes* [98].

## 5. Conclusions and Perspective

T2DM, as one of the major public health problems worldwide, is currently prevailing and seems likely to continue for some time. Therefore, there is an urgent need for new methods to prevent and treat this disease. However, most of the treatments currently in use, especially drugs with proven effects, generally focus on agents designed to directly affect signaling pathways that directly modulate the blood glucose, which usually show some side effects. However, better underlying causes of T2DM indicates that regulating the gut microbiota may be a potential way to treat this disorder.

Natural plants, especially medicine food homology and functional foods, are considered to be an ideal candidate for oral treatment because of their effective, non-toxic, few side effects, and have received widespread attention in the of management of T2DM. As described in this review, research, especially in animal models, supports this view. Additionally, studies on MFH and FF suggests that their beneficial effects on T2DM may be partly mediated by their influences on gut microbiota. In fact, approaches such as inhibiting low-grade inflammation to prevent T2DM through regulating gut microbiota have existed, but recent studies on impacts of gut microbiota suggest it may be a possible medium for preventing this disorder. In this regard, further studies on the impacts of MFH and FF on the gut microbiota are worthy of in-depth attention, in humans, paving the way for better treatment and prevention of T2DM.

## Figures and Tables

**Table 1:** Effects of ingredients from MFH and FF on T2DM via gut microbiota.

Ingredients		Source	Microbiota Findings	Mechanism	Study Types and Sequencing Method	Animals	Dose and Duration	Refs.
Saponins	Ginsenoside Rk3	Panax notoginseng	↑ *Lactobacillaceae, Helicobacteraceae, Neococcaceae, Bifidobacteriaceae* ↓ *Ratio of Firmicute to Bacteroidete*;	Inhibit the inflammatory cascade by suppressing the TLR4/NF-κB pathway	In vivo; 16S rRNA Sequencing Analysis	C57BL/6 Mice	60 mg/kg/day; 8 weeks	[26]
	20(S)-ginsenoside Rg3	*Panax ginseng* C. A. Meyer	↑ *Bacterial diversity*	Improve bacterial diversity	In vivo; principal component analysis	Male Wistar rats	20 mg/kg/day; 2 weeks	[27]
	Ginsenoside Rb1	*Panax ginseng* C. A. Meyer	Unclear	Inhibit deglycosylation in the diabetic rats	In vivo; 16S rRNA Sequencing Analysis	Male Sprague-Dawley rats	100 mg/kg/day; 72 h	[28]
	Saponin-containing Korean red ginseng extracts	Korean red ginseng (*Panax ginseng* Meyer)	↑ *Parabacteroides, Allistipes, Lactobacillus* ↓ *Barnesiella, Mucispirillum, Lactococcus, Oscillibacter, Helicobacter*	Improve IR and glucose intolerance	In vivo; 16S rRNA Sequencing Analysis	C57BL/6	235 mg/kg/day; 4 weeks	[29]
	Saponin extract of *Polygonatum sibiricum*	*Polygonatum sibiricum* (Liliaceae)	↑ *Bifidobacteria, Lactobacillus*; ↓ *Enterobacteriaceae, Enterococcus, C. perfringens*	Improve IR	In vivo; Bacteria plate count	ICR male mice	1.0, 1.5, or 2.0 g/kg/day; 5 weeks	[30]
Polysaccharides	Polysaccharides (MDG-1) from Ophiopogonis Radix	*Ophiopogon japonicus* (Thunb.) Ker-Gawl. (Liliaceae)	↑ *Lactobacillus, Bifidobacterium*; ↓ *Escherichia coli, Streptococcus*	Improve SCFAs metabolism	In vivo; 16S rRNA Sequencing Analysis	KKay mice	300 mg/kg/day; 8 weeks	[31,32]
	Homogeneous polysaccharides from crude *Lycium barbarum* polysaccharides	*Lycium barbarum* L.	↑ *Firmicutes/Bacteroides, SCFAs*	Regulate SCFAs levels	In vivo; 16S rRNA Sequencing Analysis	C57BL/6	50 mg/kg/day; 12 weeks	[33]
	*Polygonatum sibiricum* polysaccharide	*Polygonatum sibiricum* (Liliaceae)	↑ *Firmicutes, Veillonella, Escherichia-Shigella, Klebsiella*; ↓ *Proteobacteria, Bacteroides*	Regulate bacterial diversity	In vitro; 16S rRNA Sequencing Analysis	/	/	[34]
	polysaccharide-rich extracts of *A. venetum*	*Apocynum venetum*	↑ *Odoribacter, Anaeroplasma, Parasutterella, Muribaculum*; ↓ *Enterococcus, Klebsiella, Aerococcus*.	Attenuate oxidative stress and SCFAs levels	In vivo; 16S rRNA Sequencing Analysis	Male C57BL/6 J mice	400 mg/kg/day; 4 weeks	[35]
	*Maydis stigma* polysaccharides	*Zea mays subsp. mays*	↑ *Lactobacillus and Bacteroides*	Restore the intestinal microflora balance	In vivo; 16S rRNA Sequencing Analysis	Male KM mice	400, 600, 800 mg/kg/day; 5 weeks	[36]
	*Plantago asiatica* L. polysaccharides	*Plantago asiatica* L.	↑ *Colon bacterial diversity, Bacteroides vulgatus, Lactobacillus fermentum, Prevotella loescheii, Bacteroides vulgates*	Increase the levels of SCFAs	In vivo; 16S rRNA Sequencing Analysis	Wistar rats	100, 200 or 400 mg/kg/day; 5 weeks	[37]
	Pseudostellariae Radix	*Pseudostellaria heterophylla* (Miq.) Pax ex Paxet Hoffm.	↑ *Lactobacillus, Bifidobacterium*	Attenuate oxidative stress; suppress inflammatory response	In vivo; 16S rRNA Sequencing Analysis	Male C57BL/6 J	500 mg/kg/day; 4 weeks	[38]
	Polysaccharides of *Lactobacillus plantarum*-fermented Momordica charantia	*Momordica charantia* L.	↑ *Lactococcus laudensis, Prevotella loescheii, diversity of gut microbiota, SCFAs* ↓ *pH value*	Attenuate oxidative stress	In vivo; 16S rRNA Sequencing Analysis	Male Wistar rats	50, 100 mg/kg/day; 4 weeks	[39]
	mulberry fruit polysaccharide	*Morus alba* L.	↑ *Lactobacillus, Allobaculum, Bacteroides, Akkermansia, SCFA (butyrate, propionate)*. ↓ *Firmicutes, Bacillus, Lactobacillus*	Attenuate oxidative stress	In vivo; 16S rRNA Sequencing Analysis	Male *db/db* mice	500, 800 mg/kg/day; 8 weeks	[40]
	Pumpkin polysaccharide	*Cucurbita moschata* (Duch. ex Lam.)	↑ *Bacteroidetes, Prevotella, Deltaproteobacteria, Oscillospira, Veillonellaceae, Phascolarctobacterium, Sutterella, Bilophila*	Increase SCFAs production	In vivo; 16S rRNA Sequencing Analysis	Male Wistar rats	1000 mg/kg/day; 4 weeks	[41]
Flavonoids	Baicalein	*Oroxylum indicum*, Scutellaria baicalensis	↑ *Bacteroides, Bacteroidales S24-7*	Alleviate inflammation and IR	In vivo; 16S rRNA Sequencing Analysis	Male Wistar rats	50, 150 mg/kg/day; 4 weeks	[42]
Terpenoids	2β-hydroxybetulinic acid 3β-oleiate	*Euryale ferox salisb.*	Unclear	Reduce blood glucose, regulate dyslipidemia and antioxidant enzymes, protect pancreatic β-cell	In vivo	Male Wistar rats	60 mg/kg/day; 45 days	[43]
Alkaloids	Berberine	Coptidis rhizoma and *Berberis vulgaris*	↑ *Bacteroidetes, Lactobacillaceae; diversity of the gut microbiota* ↓ *Proteobacteria, Verrucomicrobia*	Alleviate inflammation via NF-κB signaling pathways	In vivo; Real-Time PCR Assay	Male Sprague-Dawley rats	200 mg/kg/day; 6 weeks	[44,45]
Others	total glycoside from R. glutinosa leaves	*Rehmannia glutinosa*	↑ *Firmicutes, norank_f_Bacteroidales_S24-7_group*	Regulate glycolipid, inhibit the expression of α-SMA, TGF-β1, Smad3 and Smad4 in the kidney tissues	In vivo; 16S rRNA Sequencing Analysis	*db/db* mice	520 mg/kg/day; 6 weeks	[46]
	low-polar S. grosvenorii glycosides	*Siraitia grosvenorii* (Swingle) C.	↑ *Elusimicrobium, Lachnospiraceae_UCG-004*	Increase SCFAs production (acetate, butyrate, and 1β-hydroxycholic acid)	In vivo; 16S rRNA Sequencing Analysis	Sprague-Dawley rats	20 mg/kg/day; 14 days	[47]
	sea buckthorn protein	*Hippophae rhamnoides* L.	↑ *Bifidobacterium, Lactobacillus, Bacteroides* ↓ *Clostridium coccoides, PH value*;	Increase intestinal microorganism diversity and SCFAs levels	In vivo; 16S rRNA Sequencing Analysis	ICR mice	50, 100 and 200 mg/kg/day; 30 days	[48]
	Long chain of inulin-type fructans	inulin	↑ *Firmicutes/Bacteroidetes ratio; Ruminococcaceae, Lactobacilli*	Regulate SCFAs levels	In vivo; 16S rRNA Sequencing Analysis	Female NOD/LtJ mice	5% diet; 24 weeks	[49]
	cinnamon oil	Cortex Cinnamomi	↑ *Bacteroides* ↓ *Clostridia flora IV*	Improve IR	In vivo; 16S rRNA Sequencing Analysis	Sprague-Dawley rats	0.384 g/kg/day; 30 days	[50,51]

Abbreviations: SCFAs, short-chain fatty acid. IR, insulin resistance. ↑, Increase. ↓, Decrease.

**Table 2:** The role and mechanism of extracts in MFH and FF on T2DM through modulating gut microbiota.

MFH/FF	Source	Microbiota Findings	Mechanism	Test Sections	Study Type and Sequencing Method	Animals	Dose and Duration	Refs.
*Fructus Aurantii Immaturus*	*Citrus aurantium* L.	↓ *Lachnospiraceae NK4A136*, *Prevo tellaceae UCG-003*, *Prevotellaceae NK3B31*, *Lachnospiraceae* UCG-008, *Ruminiclostridium* 9, *Ruminococcaceae* UCG-014; ↑ *Lactobacillus*, *Alloprevotella, Treponema 2*	Restore the intestinal microflora balance	Water extracts of fried *Fructus Aurantii Immaturus* with wheat bran decoction	In vivo; 16S rRNA Sequencing Analysis	Male Sprague-Dawley rats	9 g/kg/day; 14 d	[83]
*Atractylodes macrocephala* Koidz	*Atractylodes macrocephala* Koidz (Compositae)	↑ *Bacteroides thetaiotaomicron*, *Methanobrevibacter smithii*	Upregulate GLP-1R, PI3K, PDX-1 expressions, and suppress inflammation (decrease FOXO1, NF-κB p65)	Water extracts of *Atractylodis macrocephalae* Rhizoma (AMK)	In vivo; 16S rRNA Sequencing Analysis	*db/db* mice	100 mg/kg/day; 3 weeks	[84]
*Anemarrhena asphodeloides*	*Anemarrhena asphodeloides* Bge.	↑ *Blautia coccoides* (in vitro) ↓ *Proteobacteria*, *Facklamia, Oligella*, and *Klebsiella*	Suppress the increased oxidative stress and inflammatory activation.	Water extract of *A*. *asphodeloides*	In vivo; 16S rRNA Sequencing Analysis	Male SPF Wistar rats	20, 60, 180 mg/kg/day; 4 weeks.	[85]
*Lycium barbarum*	*Lycium barbarum* L.	↑ the ratio of *Firmicutes* to *Bacteroidetes*; ↓ *Parasutterella*, *Marvinbryantia*, *Blautia*, *Ruminococcus_1*, *Prevotellaceae_NK3B31_*group	Improve liver, kidney, and pancreas injury and regulate metabolic profiles	Water extract of *L. barbarum* leaf	In vivo; 16S rRNA Sequencing Analysis	(SPF)-grade rat	1.04, 2.08 g/kg/day; 4 weeks	[66]
*Alpinia oxyphylla* Miq.	*Alpinia oxyphylla* Miq. (Zingiberaceae)	↑ *Akkermansia*; ↓ *Helicobacter*	Modulate gut microbiota composition	Water extract of *Alpinia oxyphylla* Miq.	In vivo; 16S rRNA Sequencing Analysis	*db/db* mice	100, 300, 500 mg/kg/day; 8 weeks	[86]
Chinese propolis	Chinese propolis	↑ *Roseburia*, *Intestinimonas, Parabacteroides goldsteinii*, *Parabacteroides distasonis*; ↓ *Faecalibacterium*, *Prevotella, Bacteroides vulgatus*	Reduce inflammation	Ethanol extract of propolis	In vivo; 16S rRNA Sequencing Analysis	C57BL/6	200, 300 mg/kg/day; 12 weeks	[87,88]
*Puerariae Radix*	*Pueraria lobata*	↑ *Lactococcus, Ruminococcus*	Inhibit obesity and inflammatory-related parameters	30% ethanol extracts of dried root of *P. lobata*	In vivo; 16S rRNA Sequencing Analysis	Female C57BL/6 J mice	400 mg/kg/day; 10 weeks	[89]
Mulberry leaf	*Morus alba* L.	↑ *Bacteroidetes*, *Proteobacteria*; *Clostridia*	Improve IR	mulberry leaf powder	In vivo; 16S rRNA Sequencing Analysis	Sprague-Dawley male rats	20% (*w/w*) in diet; 13 weeks	[90]
Coicis Semen	*Coix lacryma-jobi L.* var. *ma-yuen* (Roman.) Stapf	↑ *Lactobacillus, Coprococcus, Akkermansia, Akkermansia muciniphila, Lactobacillus agilis*	Improve glucose homeostasis	*Coicis Semen* power included in diet	In vivo; 16S rRNA Sequencing Analysis	C57BL/6 mice	0.5 g/100 g; 5 weeks	[91]
*Astragali Radix*	*Astragalus membranaceus* (Fisch.) Bge. var. mongholicus (Bge.)	↑ ratio of *Firmicutes*/*Bacteroidota*; *Lactobacillales*	Regulate gut microbiota	*Astragali Radix* decoction vesicle-like nanoparticles extracted by ltracentrifugation;	In vivo; 16S rRNA Sequencing Analysis	*db/db* mice	5.3, 10.6, 21.1 g/kg/day; 3 weeks	[92]
*Dendrobium candidum*	*Dendrobium candidum* Wall Ex Lindl	↑ *Akkermansia*, *Parabacteroides*	Improve glucose intolerance and IR	*Dendrobium officinale* extract	In vivo; 16S rRNA Sequencing Analysis	T2D mice	1.0 g/kg/day; 30 days	[93]
hemp seed	*Cannabis sativa* L.	↑ *Bacteroidetes*; ↓ *Firmicutes*	Modulate gut microbiota	hemp seed oil-water mixture	In vivo; 16S rRNA Sequencing Analysis	Female KM mice	0.2, 0.4 mL; 10 days	[94]
*Dioscoreae* Rhizoma	*Dioscorea opposita* Thunb.	↑ *Bifidobacterium, Adolescentis, Bifidobacterium infantis*	Modulate gut microbiota	yam gruel	In vivo; 16S rRNA Sequencing Analysis	Human patients	150 g/day; 3 months	[95]

Abbreviations: IR, insulin resistance. ↑, Increase. ↓, Decrease.

**Table 3:** The role and mechanism of formula extracts in MFH and FF on T2DM through modulating gut microbiota.

MFH and FF	Microbiota Findings	Mechanism	Test Sections	Study Type and Sequencing Method	Animals	Dose and Duration	Ref.
Wumeiwan	↓ *Bacteroidetes*, *Actinobacteria*, *Bacteroides, Clostridium*; ↑ *Firmicutes*, *DeltaProteobacteria*, *Lactobacillus*	Improve SCFA, inhibit inflammatory mediums (TNF-α, IL-10)	Decoction concentrate	In vivo; 16S rRNA Sequencing Analysis	Sprague-Dawley rats	5, 10, 20 g/kg/day; 4 weeks	[97]
Daesiho-Tang	↑ *Bacteroidetes, Bacteroidetes/Firmicutes ratio, Akkermansia Bifidobacterium, Lactobacillus*; ↓ *Firmicutes*	Modulate intestinal microbiota	Water extracts	In vivo; 16S rRNA Sequencing Analysis	Male C57BL/6 mice	700 mg/kg/day; 12 weeks	[98]
Gegen Qinlian Decoction	↑ *Lactobacillus johnsonii, Stomatobaculum longum strain* ACC2, *Bacteroides vulgatus*	Suppress inflammation: reduce the levels of LPS, TNF-α, IL-6	Crude drugs	In vivo; 16S rRNA Sequencing Analysis	KK-Ay mice	4.44, 13.30, 40.00 g/kg/day; 4 weeks	[99]
A mixture of *D. officinale* and American *ginseng*	↑ ratio of *Bacteroidetes to Firmicutes*, *Prevotella*, *Akkermansia*; and SCFA-producing bacteria; ↓ *S24-7*/*Rikenella*/*Escherichia coli*.	Decrease inflammation (IL-6 and TNF-α) and oxidative stress; improve intestinal flora balance	Mixture of *D. officinale* and American ginseng	In vivo; 16S rRNA Sequencing Analysis	Dogs	160 mg/kg/day; 60 days	[100]
*Chinese Herbal Formula Shenzhu Tiaopi* *Granule*	↑ *Lactobacillus*; ↓ *Firmicutes*/*Bacteroidetes* ratio, *Bacteroidetes, Allobaculum*, *Desulfovibrionaceae*	Inhibit inflammation, ameliorate IR	Shenzhu Tiaopi Granule	In vivo; 16S rRNA Sequencing Analysis	Male Goto-Kakizaki (GK)	1000 mg/kg/day; 8 weeks	[101]
*Qijian Mixture*	↑ *Bacteroidetes*	Inhibit inflammation and oxidative stress	Qijian Mixture	In vivo; 16S rRNA Sequencing Analysis	Male KKay mice	1.795, 5.385 g/kg/day; 5 weeks	[102]
*Anemarrhena asphodeloides* Bge.and *Phellodendron chinense* Schneid	↓ *Bacteroidetes*; *Bacilli*, *Lactobacillus* ↑ *Firmicutes, Proteobacteria*; *Clostridia,* *Romboutsia*, *Bacteroides*	Improve intestinal microbiota	Decoction concentrate	In vivo; 16S rRNA Sequencing Analysis	Sprague-Dawley rats	6.48 g/kg/day; 30 days	[103]
Combination of Aronia, Red Ginseng, Shiitake Mushroom and Nattokinase	↓ *Clostridales*; ↑ *Bacterioidales*	Improve IR	Water extracts of the combination	In vivo; 16S rRNA Sequencing Analysis	Sprague Dawley rats	0.5, 1.0 g/kg/day; 12 weeks	[104]
*Scutellaria baicalensis* Georgi, SR and *Coptis chinensis* Franch, CR	↑ SCFAs-producing bacteria: *Bacteroidales S24-7 group_norank, Eubacterium nodatum group, Parasutterella, Prevotellaceae UCG-001, Ruminiclostridium, Ruminiclostridium * ↓ Secondary bile acid-producing bacteria *Escherichia Shigella*;	Increase microbially derived SCFAs	Water extracts	In vivo; 16S rRNA Sequencing Analysis	Male Sprague-Dawley rats	6.3 g/kg/day; 1 month	[105]

Abbreviations: IR, insulin resistance. ↑, Increase. ↓, Decrease.
